# Hormone Replacement Therapy: An Increased Risk of Recurrence and Mortality for Breast Cancer Patients?

**DOI:** 10.6004/jadpro.2015.6.4.3

**Published:** 2015-07-01

**Authors:** Molly Lupo, Joyce E. Dains, Lydia T. Madsen

**Affiliations:** MD Anderson Cancer Center, Houston, Texas

## Abstract

Historically, randomized controlled trials (RCTs) have shown an increased risk of recurrence and mortality among women who have used primarily oral HRT after breast cancer. However, many of these studies have had design flaws that may impact the findings. Numerous investigators have concluded that additional RCTs should be performed, but because of ethical issues and logistic challenges, large-scale RCTs are unlikely. Thus, the authors conducted an integrative review investigating recurrence and mortality data among breast cancer survivors who have used hormone replacement therapy (HRT). They recommend a stepwise algorithm for treating vaginal symptoms in breast cancer survivors: (1) start with nonhormonal treatments; (2) progress to a detailed discussion among patients and health-care professionals about the current known risks and benefits of vaginal estrogen; and (3) conclude with mutual decision-making between health-care providers and patients regarding the use of vaginal estrogen treatment.

More than a quarter of a million cases of breast cancer are diagnosed each year in the United States. Of these cases, 75% are estrogen receptor (ER)–positive or progesterone receptor (PR)–positive (Esserman & Joe, 2013). The treatment regimen for receptor-positive breast cancer often includes endocrine therapy, which places these women at an increased risk for vaginal dryness, dyspareunia, urogenital atrophy, and sexual dysfunction. The gold standard of treatment for these symptoms is hormone replacement therapy (HRT); however, many health-care providers question the safety of HRT and its relationship to disease recurrence.

## BACKGROUND

Endocrine therapy slows or inhibits the proliferation of hormone-sensitive tumors by blocking the body’s ability to produce hormones. Women with receptor-positive breast cancer who are treated with these agents may subsequently experience menopausal symptoms, which can be divided into two categories: systemic or local.

Systemic symptoms routinely seen include hot flashes, insomnia, and mood changes. Local symptoms include urogenital atrophy and vaginal dryness (Casper, Barbieri, Crowley, & Martin, 2015). While health-care providers attempt to manage menopausal symptoms, they must try to do so without increasing the risk for recurrence. Although endocrine therapy has improved disease-free and overall survival, the consequent acceleration of menopause may negatively impact quality of life (Pritchard, 2014; Trinkaus, Chin, Wolfman, Simmons, & Clemons, 2008).

A higher incidence of osteoporosis, vasomotor symptoms, and gynecologic symptoms has been associated with endocrine therapy (Ruddy & Partridge, 2014). Women treated with endocrine therapy are at an increased risk for vaginal dryness, dyspareunia, urogenital atrophy, and sexual dysfunction. Dyspareunia and urogenital atrophy increase discomfort and pain during sexual activity. Interestingly, Pritchard (2014) as well as Ruddy and Partridge (2014) have reported a relationship between sexual dysfunction and depression in this population, suggesting that women receiving endocrine therapy are at risk for multiple comorbidities.

Historically, researchers have argued against using HRT to treat the gynecologic symptoms associated with endocrine therapy in breast cancer survivors. However, local HRT is the standard treatment to control and alleviate vaginal symptoms. Many health-care providers are hesitant to prescribe local HRT to women with breast cancer out of concern that it may increase the risk for recurrence (Trinkaus et al., 2008).

A review of the medical literature from the 1990s (Verheul et al., 2000) shows mixed findings regarding an association between HRT and breast cancer recurrence. Although the data did not exclude HRT as a contributing factor in breast cancer recurrence, it did not prove an increased risk with its use.

One of the two randomized controlled trials (RCTs; Col, Kim, & Chlebowski, 2005) cited Marsden, Whitehead, A’Hern, Baum, and Sacks (2000) reported numerically more breast cancer recurrences among HRT users, although this was not statistically significant. Another RCT, the widely publicized Women’s Health Initiative trial, raised concerns about the use of HRT for breast cancer survivors (Rossouw et al., 2002). Another study noted that physicians’ attitudes toward HRT contribute to patients’ reluctance to use it (Trinkaus et al., 2008).

This article extends the review by Verheul et al. (2000) and presents an integrative examination of research on breast cancer recurrence and mortality associated with HRT, primarily from the year 2000. Examination of the delivery methods and dosages of HRT may offer a better understanding of the impact of HRT among breast cancer survivors. This integrative review of findings is derived from RCTs, prospective studies, and retrospective studies. We propose that a comprehensive perspective may provide updated evidence regarding the use of HRT in breast cancer survivors (Whittemore & Knafl, 2005).

## METHODS

Electronic databases were searched, including Medline (Ovid), Embase, and Scopus. The following search terms were used: estrogen replacement therapy, hormone replacement therapy, HRT, local hormonal replacement, local hormone replacement, vaginal estrogen, local estrogen, breast neoplasms, recurrence, neoplasm recurrence local, survivors, and survivor. English only was selected for Medline Ovid but not for Embase or Scopus. The time period searched was from January 1999 to February 2014.

A total of 624 references were found, and 467 articles were excluded, as they were not relevant based on the title of the paper. After closer examination, an additional 143 sources were excluded for the following reasons: evaluated nonbreast cancers (5); consisted of literature and/or systematic reviews (40); consisted of a case report (1); consisted of duplicate data (4); consisted of letters (17); consisted of evaluating lipids and skeletal health (1); consisted of pregnancy data (1); an opinion review (1); did not include recurrence or mortality information (30); consisted of a nonhormonal treatment (1); article was not in English (2); consisted of nonclinically relevant information (9); consisted of only premenopausal women’s data (1); consisted of a survey (1); consisted of primarily symptom management (16); consisted primarily of testosterone and/or androgen treatment (2); used primarily a synthetic steroid as the treatment (8); and the article was no longer available (2). One article was included after it was located in the reference section of one of the included articles (Holmberg et al., 2008).

This integrative review consists of 14 articles, representing a mixture of RCTs, prospective studies, and retrospective studies. Multiple and diverse research methodologies were purposely included for a broad examination of the current evidence related to this complex topic (Whittemore & Knafl, 2005).

## RESULTS

The following data are organized into an overall assessment of HRT (recurrence and mortality), followed by a synthesis of the impact of (1) oral vs. vaginal HRT; (2) hormone receptor–positive vs. hormone receptor–negative cancer; and (3) treatment duration. Identification of the relevant literature and values of statistically significant relative ratios (RR) and hazard ratios (HR) are shown in Table 1. For further delineation between the studies that used oral vs. local HRT, see Table 2. Because Holmberg et al. (2008) is an extension of Holmberg et al. (2004), which was a research letter, only the results of Holmberg et al. (2008) are described in this article.

**Table 1:**
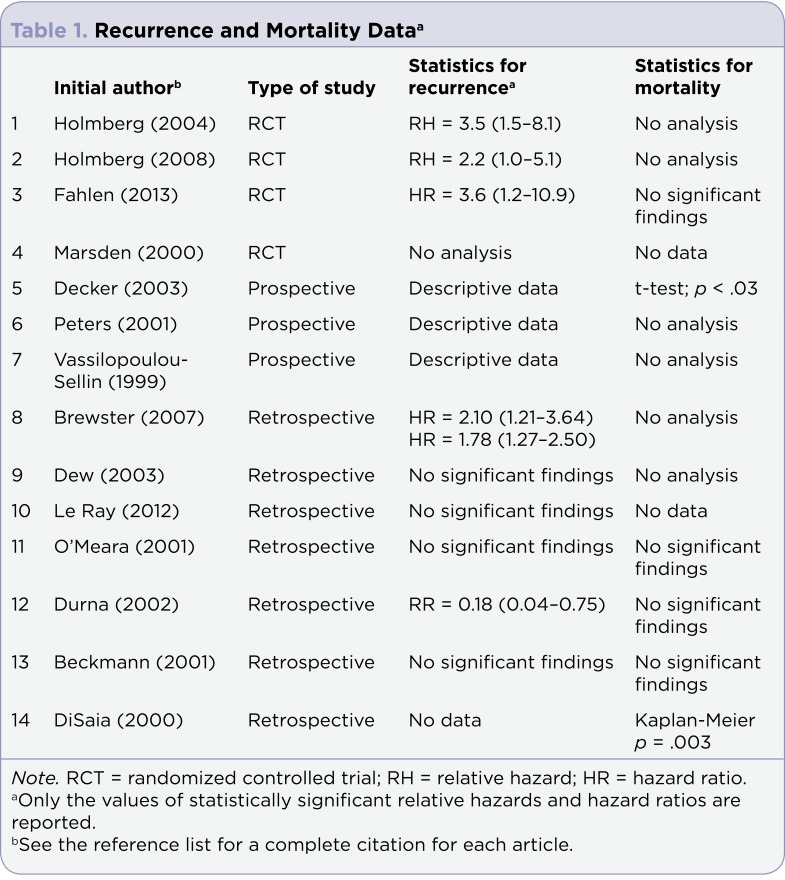
Recurrence and Mortality Data^a^

**Table 2:**
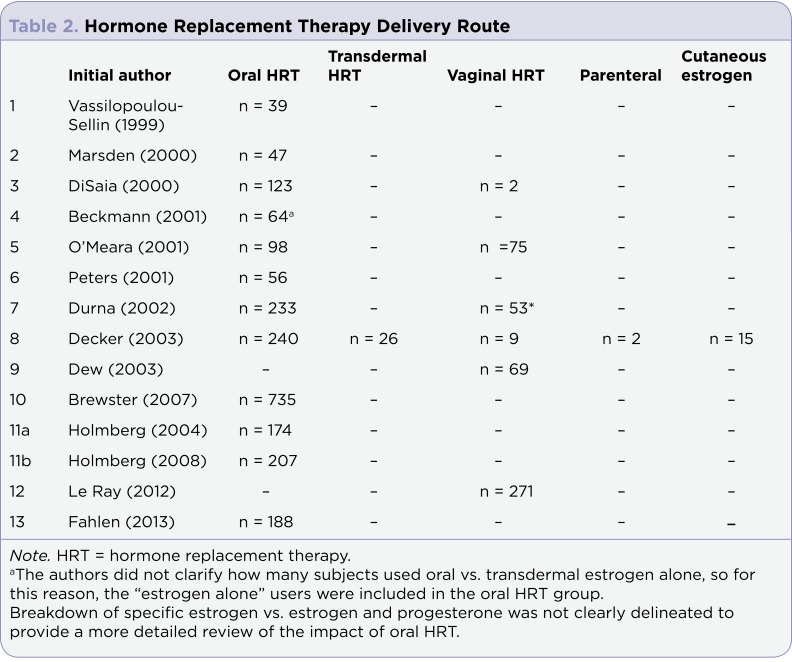
Hormone Replacement Therapy Delivery Route

**Analysis of Recurrence and Mortality**

*Recurrence*: Two RCTs reported a significantly increased risk of breast cancer recurrence. In a study of 442 women with a history of breast cancer, Holmberg et al. (2008) reported a recurrence rate of 17.6% among those who used primarily oral HRT (mean duration, 24 months; 4.1 years of follow-up). Nonusers of HRT, in contrast, had a recurrence rate of 3.2%. The adjusted HR was 2.2 (1.0–5.1).

Fahlen et al. (2013) followed 378 women treated for breast cancer for a median of 10.8 years. A total of 188 of the women reported using oral HRT for a mean duration of 2.6±1.2 years, whereas women in the non–HRT group were allowed to use local vaginal treatment with low-dose estrogen gels or vaginal suppositories. Fahlen et al. (2013) reported a significantly increased risk for first-event recurrence, but only in the contralateral breast (HRT users 7.4% vs. non–HRT users 2.1%), with an HR of 3.6 (1.2–10.9). They attributed the increased recurrence rate to higher progesterone exposure in women who used combination estrogen and progesterone as opposed to those who received estrogen alone.

There may be several reasons for the difference in findings reported by Fahlen et al. (2013) and Holmberg et al. (2008). Each focused on different outcome variables (percentage of patients vs. type of first event, respectively). Fahlen et al. (2013) noted the significant heterogeneity between the two studies and suggested that population variation between the two samples may have played a role. Fahlen et al. (2013) provided a longer median follow-up (10.8 years) than did Holmberg et al. (2008; 4.1 years), suggesting that length of follow-up might be associated with recurrence outcomes.

Furthermore, both studies reported challenges in recruitment and compliance. Holmberg et al. (2008) reported compliance issues, with only 88% of patients in the HRT arm taking HRT, and Fahlen et al. (2013) reported that only 77% of those in the treatment group were compliant. In addition, Holmberg et al. (2008) reported that many women took HRT for more than the 2 years specified by the protocol and that 10% of those in the non-HRT group had taken some form of HRT after inclusion in the trial.

Although the studies by Fahlen et al. (2013) and Holmberg et al. (2008) used an RCT design, both had limitations. Neither study was blinded nor used controls without confounding variables (i.e., heterogeneity of the sample, duration of follow-up, compliance failure, and exposure of some non-HRT patients to HRT). In addition, early termination of the studies increased the likelihood of a selection bias. Finally, these studies have limited external validity (i.e., findings may not be generalizable because the samples comprised exclusively Swedish women).

In contrast to these findings, Brewster et al. (2007) obtained medical records from 2,327 women with early-stage breast cancer and 735 women who had used oral HRT. They reported a significantly higher recurrence-per-patient risk among non-HRT patients (19.4%) vs. HRT patients (9.8%), with an HR of 1.78 (1.27–2.50). This study used a Cox proportional hazard regression model adjusted for relevant extraneous variables (e.g., year of diagnosis and race).

The remaining observational studies (Beckmann et al., 2001; Dew, Wren, & Eden, 2003; Le Ray, Dell’Aniello, Bonnetain, Azoulay, & Suissa, 2012; O’Meara et al., 2001) found no statistical evidence to support an increased risk of breast cancer recurrence from HRT. Lea et al. (2004) stated, "However crude and open to treatment bias, these (observational) data do not demonstrate HRT to be associated with an increased recurrence of breast cancer." None of the studies that used prospective designs (Decker et al., 2003; Peters, Fodera, Sabol, Jones, & Euhus, 2001; Vassilopoulou-Sellin et al., 1999) reported statistical analyses of recurrence data.

*Mortality*: Two studies reported statistically significant analyses for mortality data that favored hormone therapy (Decker et al., 2003; DiSaia, Brewster, Ziogas, & Anton-Culver, 2000). Decker et al. (2003) followed 277 breast cancer survivors who received estrogen replacement therapy (ERT) for a mean duration of 3.7 years and then for 7.75±4.96 years. The investigators used a variety of ERTs, including oral, transdermal, vaginal, parenteral, and cutaneous, and found a significantly greater risk in the mean time to death for the non-ERT vs. ERT users of 9.85 and 8.15 years, respectively (*p* < .03).

DiSaia et al. (2000) studied 125 patients with breast cancer who used primarily oral HRT (n = 123) vs. vaginal estrogen (n = 2) for a median of 22 months; they found a significant survival advantage between the HRT and non–HRT users of 88% and 63%, respectively (*p* = .003). Both studies were observational and used a Kaplan-Meier survival analysis. However, the studies applied different outcome variables regarding time to death (Decker et al., 2003) vs. survival advantage (DiSaia et al., 2000).

Among the studies that reported statistical analyses for mortality, one of three was an RCT (Fahlen et al., 2013), and four of seven were retrospective studies (Beckmann et al., 2001; DiSaia et al., 2000; Durna et al., 2002; O’Meara et al., 2001). Only one of the prospective studies reported statistical analyses of mortality data (Decker et al., 2003). None of the other studies cited in this integrative review reported statistical analyses for overall mortality. Other studies did not analyze mortality because of a lack of data and/or insufficient sample sizes.

**Oral vs. Vaginal HRT**

Fahlen et al. (2013) treated patients in the HRT group primarily with oral estrogen, whereas patients in the non-HRT group were treated with low-dose estrogen gels or vaginal suppositories. Among oral HRT users, the incidence of cancer recurrence in the contralateral breast was 7.4% vs. 2.1% for non-HRT users (HR = 3.6 [1.2–10.9]). The HR for mortality among patients in the oral HRT vs. non-HRT groups was not statistically significant. Unfortunately, the amount of estrogen exposure in the non-HRT group could not be precisely determined; as a result, a definitive statement about oral vs. vaginal use is not possible.

Durna et al. (2002) compared women who used different types of HRT. When they compared patients who used vaginal estrogen with non-HRT patients, the authors reported a significantly lower risk of recurrence of new breast cancer for vaginal estrogen users (9.1%) vs. non-HRT users (29.5%), with a RR of 0.18 (0.04–0.75). Neither Durna et al. (2002) nor Fahlen et al. (2013) found evidence of a statistically significant increased mortality risk among patients using local vaginal hormone therapy.

Some of the women (n = 69, or 4.7%) in the Dew et al. (2003) study used low-dose topical vaginal estrogen cream and vaginal estrogen tablets, whereas the others used a form of hormonal therapy or not. Although not statistically significant, 9% of the patients treated with topical vaginal estrogen experienced tumor recurrence vs. 22.4% in the nontopical vaginal estrogen group. Dew et al. (2003) did not specify the type or location of the tumors.

Le Ray et al. (2012) compared women taking local hormonal therapy with women who had been on hormonal treatment but not while on treatment-related endocrine therapy. The researchers found no statistically significant increased risk of recurrence for women on local hormone therapy. Dew et al. (2003) and Le Ray et al. (2012) reported no data and conducted no statistical analyses, respectively, for mortality.

**Hormone Receptor–Positive vs. Hormone Receptor–Negative Patients**

As detailed previously, Brewster et al. (2007) reported the risk of recurrence by patient populations when compared by various combinations of ER and PR status. Patients who had never used HRT had no increased risk of recurrence based on their ER and PR status. However, for the cohort that had used HRT, those in the ER- and PR-negative groups had a statistically significant increased risk of recurrence of 25% compared with patients in the ER- and PR-positive groups, whose risk of recurrence was 10%, with an HR of 2.10 (1.21–3.64). No direct comparison of HRT and non-HRT groups based on hormone-receptor status was included, and the authors did not report mortality data.

Peters et al. (2001) also assessed groups in accordance with specific ER and PR status. Recurrence and mortality data were descriptive; no risk analyses were reported because of small sample sizes. No other studies in this integrative review reported stratification data relevant to hormone-receptor status.

**Duration of Treatment**

Direct comparisons among studies associated with the duration of treatment were not possible because none of the studies examined HRT treatment as a function of duration of treatment. However, indirect comparisons are possible by examining the mean (or median) durations of treatment among studies for increased and decreased risk of recurrence or mortality.

In general, studies reporting increased risks of recurrence or mortality (Decker et al., 2003; Fahlen et al., 2013; Holmberg et al., 2008) had longer durations of treatment (24–42 months) than did studies reporting decreased risks of recurrence and mortality (12–22 months; DiSaia et al., 2000; Durna et al., 2002). The results suggest that longer exposure to HRT increases risk, but the role of duration of treatment requires further systematic exploration.

## DISCUSSION

The tendency among investigators to rely solely on RCTs to evaluate the risks associated with HRT is widespread. Many investigators express caution about recommending HRT until data from RCTs demonstrate its safety (Dew et al., 2003; Durna et al., 2002; O’Meara et al., 2001; Peters et al., 2001; Trinkaus et al., 2008; Vassilopoulou-Sellin et al., 1999). Randomized controlled trials are regarded as a "gold standard," and their strengths include heightened internal validity and control over interventions. These studies are preferred when assessing a study’s design and quality; however, RCTs may not be an appropriate, feasible, or realistic design, depending on the logistics and health outcome under investigation. Additionally, RCTs occur at the expense of external validity (Oswald & Price, 2006).

In contrast, prospective and retrospective designs can (1) evaluate multiple outcomes to appraise the evidence; (2) analyze a sequence of events; and (3) statistically control for the influence of extraneous variables. Examples of observational research, such as studies conducted to examine the relationship between cigarette smoking and asthma, cancer, chronic obstructive pulmonary disease, diabetes, heart disease, and stroke, provide a strong foundation for subsequent health provider recommendations. Finally, RCTs that are poorly designed may not outweigh the merits of well-designed and well-executed observational studies. Although RCTs might be the ideal, ethical and logistical limitations may support the use of observational designs.

Collectively, the results of RCTs suggest caution in the use of orally administered HRT. However, the study by Holmberg et al. (2004 and 2008) revealed several limitations. The study was terminated early and provided only descriptive data about mortality (Holmberg et al., 2008). Furthermore, for adequate power, the Holmberg et al. (2004 and 2008) study specified a total sample size of 1,300 women in the non-HRT group, but only 171 were included. Finally, the study was not blinded.

Fahlen et al. (2013) reinforced the belief about the risk for contralateral breast cancer recurrence but not an increased risk for mortality among breast cancer survivors. This study did not specify whether it was blinded. A limitation to the Fahlen et al. (2013) study was that the authors did not specify how many members of the non-HRT group were allowed to use vaginal estrogen and for how long.

Although the Marsden et al. (2000) study was an RCT, the duration of HRT and the follow-up were limited. Moreover, the researchers reported only descriptive data with limited sample sizes in which breast cancer recurred in only two patients receiving HRT and in one patient not receiving HRT.

The mixed evidence regarding HRT and mortality is cause for cautious optimism. Findings by Decker et al. (2003) suggest that ERT use may increase longevity. Findings by DiSaia et al. (2000) found an increased survival advantage for HRT users, although the HRT user sample size was quite small. The remainder of the studies recorded no data, no analyses, and no statistically significant increased risk of mortality.

Findings from the retrospective studies of Durna et al. (2002) and Le Ray et al. (2012) suggest that local vaginal therapy may not place breast cancer survivors at a greater risk of breast cancer recurrence. In fact, the findings of Durna et al. (2002) indicated a significant reduction in the risk of recurrence, and the study by Le Ray et al. (2012) found no significant difference in recurrence risk associated with local vaginal therapy. Finally, Dew et al. (2003) and Durna et al. (2002) found no significant difference in mortality risk associated with local vaginal therapy. Le Ray et al. (2012) reported no data regarding mortality.

Although the three prospective studies (Decker et al., 2003; Peters et al., 2001; Vassilopoulou-Sellin et al., 1999) reported descriptive data for breast cancer recurrence, none provided statistical analyses. However, Decker et al. (2003) found a statistically significant increased mortality risk for ERT patients when compared with the control group.

Studies by Brewster et al. (2007) and Peters et al. (2001), from which indirect evidence is available about hormone-receptor status, preclude conclusions about ERT’s role in increasing or decreasing breast cancer recurrence or mortality risk. Similarly, the collective evidence from several studies (Decker et al., 2003; DiSaia et al., 2000; Fahlen et al., 2013; Holmberg et al., 2008) concerning the role of treatment duration provides only indirect and uncertain conclusions regarding whether this factor increases or decreases breast cancer recurrence or mortality risk. Thus, we recommend that investigators pursue future research on the role of hormone-receptor status and the duration of treatment.

This integrative review was conducted to help assess an important and common clinical practice problem by looking at heterogeneous populations and the use of established medical interventions. Such an approach may offer insights into clinical problems that an RCT might not (Yang et al., 2010).

The studies reviewed in this article primarily used oral HRT in treatment groups (Beckmann et al., 2001; Brewster et al., 2007; Decker et al., 2003; Dew et al., 2003; DiSaia et al., 2000; Fahlen et al., 2013; Holmberg et al., 2004, 2008). However, there was considerable variability in the dosage, types of estrogen used, and duration of use among the studies; even within a single study, there was significant variability.

Although vaginal or local HRT was less commonly used in these studies, a much lower dose than the oral HRT was often used (Dew et al., 2003; Durna et al., 2002; Le Ray et al., 2012). For example, a low-dose vaginal estrogen tablet delivers 1.14 mg annually, compared with a standard oral estradiol tablet, which delivers 182.5 mg annually (Pruthi, Simon, & Early, 2011).

Guyatt, Rennie, Meade, and Cook (2008) distinguished between the roles of clinical and statistical significance by arguing that clinical significance relies on the size of the effect and not merely statistical significance. In short, statistical significance may not imply clinical significance. Given the value of clinical significance, the results of this integrative review argue that the collective findings of RCTs, prospective, and retrospective designs fail to demonstrate compelling evidence of an increased risk of breast cancer recurrence or mortality associated with HRT, particularly for topical or local vaginal therapy.

## CONCLUSION

Limited and conflicting evidence exists regarding the risks associated with the use of HRT: (1) for oral and vaginal applications; (2) in the ER- and/or PR-positive breast cancer populations; and (3) for different durations of treatment (Brewster et al., 2007; Durna et al., 2002; Fahlen et al., 2013; Holmberg et al., 2008). Studies reviewed in this article, which have given limited consideration to quality-of-life issues and the role of patients and health-care providers in the decision-making process for using HRT, also add to the difficulty of making patient recommendations. Although recurrence and mortality rates are key considerations, quality of life is also a salient issue among the majority of breast cancer patients in their posttreatment lives.

Women who experience vaginal atrophy symptoms related to therapy should be counseled about their options for symptom management. For a step-wise approach to treating vaginal atrophy–like symptoms in breast cancer patients, see the article by Trinkaus et al. (2008). The option of local HRT should be presented to patients after they have exhausted other treatments for vaginal symptoms and should be accompanied by a discussion of the local risks and benefits of HRT. Together, patients and health-care professionals can evaluate those risks and benefits so patients can make informed decisions about whether or not HRT is right for them.
